# Association of Serum MiR-142-3p and MiR-101-3p Levels with Acute Cellular Rejection after Heart Transplantation

**DOI:** 10.1371/journal.pone.0170842

**Published:** 2017-01-26

**Authors:** Ihdina Sukma Dewi, Zsuzsanna Hollander, Karen K. Lam, Janet-Wilson McManus, Scott J. Tebbutt, Raymond T. Ng, Paul A. Keown, Robert W. McMaster, Bruce M. McManus, Olof Gidlöf, Jenny Öhman

**Affiliations:** 1 Department of Cardiology, Skåne University Hospital, Lund University, Lund, Sweden; 2 Prevention of Organ Failure (PROOF) Centre of Excellence, Vancouver, Canada; 3 UBC James Hogg Research Centre, Vancouver, Canada; 4 netCAD, Canadian Blood Services, Vancouver, Canada; 5 Department of Medicine, University of British Columbia, Vancouver, Canada; 6 Centre for Heart Lung Innovation, University of British Columbia, Vancouver, Canada; 7 Department of Computer Science, University of British Columbia, Vancouver, Canada; 8 Vancouver General Hospital, Vancouver, Canada; 9 Vancouver Coastal Health Research Institute, Vancouver, Canada; 10 Department of Pathology and Laboratory Medicine, University of British Columbia, Vancouver, Canada; Institut de Pharmacologie Moleculaire et Cellulaire, FRANCE

## Abstract

**Background:**

Identifying non-invasive and reliable blood-derived biomarkers for early detection of acute cellular rejection in heart transplant recipients is of great importance in clinical practice. MicroRNAs are small molecules found to be stable in serum and their expression patterns reflect both physiological and underlying pathological conditions in human.

**Methods:**

We compared a group of heart transplant recipients with histologically-verified acute cellular rejection (ACR, n = 26) with a control group of heart transplant recipients without allograft rejection (NR, n = 37) by assessing the levels of a select set of microRNAs in serum specimens.

**Results:**

The levels of seven microRNAs, miR-142-3p, miR-101-3p, miR-424-5p, miR-27a-3p, miR-144-3p, miR-339-3p and miR-326 were significantly higher in ACR group compared to the control group and could discriminate between patients with and without allograft rejection. MiR-142-3p and miR-101-3p had the best diagnostic test performance among the microRNAs tested. Serum levels of miR-142-3p and miR-101-3p were independent of calcineurin inhibitor levels, as measured by tacrolimus and cyclosporin; kidney function, as measured by creatinine level, and general inflammation state, as measured by CRP level.

**Conclusion:**

This study demonstrated two microRNAs, miR-142-3p and miR-101-3p, that could be relevant as non-invasive diagnostic tools for identifying heart transplant patients with acute cellular rejection.

## Introduction

The main goal of post heart transplantation care is to prevent allograft rejection while minimizing the dose of immunosuppressive treatment. Endomyocardial biopsy represents the gold standard for diagnosing and monitoring acute cellular rejections (ACR), but this invasive technique represents a burden and a risk to cardiac transplant patients worldwide. Sampling error, inter-observer variability and potential complications are other clinical concerns associated with this procedure[1–4]. Although some advancement has been made to find the non-invasive diagnostic tools, they are not widely used and do not eliminate the need for endomyocardial biopsy[5]. Identifying non-invasive and reliable biomarkers for early detection of acute cellular rejection is of great importance and has become a major challenge in solid organ transplantation[6,7].

In the field of biomarker discovery, there has been a growing interest in using microRNAs, small non-coding RNAs that regulate gene expression, as biomarkers in bodily fluids. The ability to accurately and rapidly detect microRNAs in biofluids combined with their tissue- and disease-specific expression make these molecules excellent biomarker candidates. Several studies have indicated specific microRNAs as useful biomarkers across different pathological conditions[8–10]. In a previous pilot study, using samples from heart transplant patients treated at Skane University Hospital (Lund, Sweden), we demonstrated proof-of-principle that the profile of serum microRNAs is altered during ACR and that miR-142-3p can discriminate significantly between histologically-verified normal and diseased states[11]. In this study we assessed the levels of of seven microRNAs that were increased in serum during ACR in our previous study, in a larger, independent cohort from the Prevention of Organ Failure (PROOF) Centre of Excellence (Vancouver, Canada). The results showed that the levels of these seven microRNA (miR-142-3p, miR-101-3p, miR-424-5p, miR-27a-3p, miR-144-3p, miR-339-3p and miR-326) were significantly higher in the ACR group compared to the control group and that each microRNA could discriminate between patients with and without ACR. MiR-142-3p and miR-101-3p had the best diagnostic performance among the seven microRNAs tested, making them the potential candidates as non-invasive biomarkers for ACR surveillance post heart-transplantation.

## Materials and Methods

### Patients and serum samples

All heart transplant recipients included in this study were enrolled as part of the Biomarkers in Transplantation Canada-wide Trial from 6 Canadian heart transplant centers (QE II Health Sciences Centre, Halifax, NS; Libin Cardiovascular Institute of Alberta, Calgary, AB; St. Boniface General Hospital, Winnipeg, MB; University of Ottawa Heart Institute, Ottawa, ON; Toronto General Hospital, Toronto, ON; St. Paul’s Hospital, Vancouver, BC), who underwent heart transplantation between February 2009 and September 2013. All participants of this study provide their verbal and written informed consent and each local research ethics board approved the study. None of the transplant donors were from a vulnerable population and all donors or next of kin provided written informed consent that was freely given. The ethics approval for that study has the following number at the University of British Columbia: H04-50286. A group of 30 heart-transplanted patients with histologically verified ACR was compared with a control group of 50 heart-transplanted patients without allograft rejection (NR) from the same centers and within the same time-period, matched by ISHLT (International Society for Heart and Lung Transplantation) 2004 classification biopsy grade, age, sex and post-transplantation of sample collection. In the post analysis quality control of the samples, 4 samples from ACR group and 13 samples from the NR group were excluded due to hemolysis and cellular contamination in the serum samples, which left 26 and 37 samples of ACR and NR, respectively, to be further analyzed. All biopsies were blindly reviewed by three expert pathologists. ACR was defined as biopsy rated as ≥1R and no rejection as 0R according to International Society for Heart and Lung Transplantation (ISHLT) 2004 classification, based on blinded review of expert pathology panel. NR subjects were matched to ACR subjects based on time post-transplant of sample collection, as well as the age and sex of the recipient.

### MicroRNA isolation from serum

Briefly, we followed RNase-free protocols throughout all procedures up to qPCR setup (after RNA has been converted to cDNA). Blood samples were collected in 4.0 ml serum tubes (VWR Cat. No. CABD 367812) and were spun down in a refrigerated centrifuge within 2 hours of collection. Using a transfer pipette, the serum was removed into a cryogenic vial and gently mixed by drawing the serum up and down in the tube several times. Once mixed, the serum was aliquoted into six 1.2 ml-cryogenic vials. The aliquots of serum were stored in -80 C freezer until selected for analysis. RNA purification was conducted on 200 μl of serum samples. Total RNA was isolated using the miRNeasy Mini Kit (Qiagen, Germany) according to the manufacturer’s instructions. Due to the low serum RNA yield, 1 μg MS2 carrier RNA (Roche, Switzerland) was added during RNA purification steps in order to maximize the RNA yield and minimize purification efficiency variation.

### cDNA synthesis

First strand cDNA was synthesized from 8 μl of eluted serum RNA in 40 μl reverse transcription reaction using miRCURY LNA^™^ Universal RT microRNA PCR System (Exiqon, Denmark) according to protocol.

### Quantitative PCR

SYBR green-based real-time quantitative PCR reactions were conducted from cDNA synthesis using Pick and Mix microRNA PCR Panel (Exiqon, Vedbaek, Denmark) according to the manufacturer’s instuctions. The qPCR amplifications were carried out by incubation for 10 minutes at 95°C, followed by 40 amplification cycles at 95°C for 10 sec and 60°C for 1 min. All reactions were performed in duplicate. Each panel consists of two 96-well PCR plates with pre-aliquoted LNA™ enhanced primer sets for miR-142-3p, miR-326, miR-144-3p, miR-101-3p, miR-27a-3p, miR-339-3p, miR-424-5p, miR-451, miR-23a. Delta Ct of (miR-23a-3p#x2014;miR-451) was used as a control for cellular contamination and hemolysis[12], where values of >5 is an indicator of possible erythrocyte microRNA contamination affecting the data obtained in human samples. A mix of synthetic RNA spike-in, Uni Sp2, UniSp6 and UniSp3 IPC (Exiqon, Vedbaek, Denmark) were added as control to monitor the efficiency of the RNA isolation, cDNA synthesis and qPCR steps, respectively and included in each Pick and Mix microRNA PCR panels. Amplification was performed using a StepOnePlus Real-Time PCR System (Applied Biosystems). Data analysis was performed using Exiqon GenEx Software 2.0. Briefly, raw data was normalized for run-to-run variations using UniSP3 IPC as an inter-plate calibrator, and a cut off for unspecific amplification was set at C_t_ = 37. Relative expression was calculated using the 2^-ΔΔCt^ method and UniSp6 spike-in was used as reference for normalization.

### Statistical analysis

GraphPad Prism 6.0 software (GraphPad Software Inc., San Diego, USA) and SPSS Statistics v.22.0 (IBM Corp., Armonk, USA) were used to perform statistical analysis. The normality test used was D’Agostino-Pearson omnibus test. It could in our case not detect a deviation from a Gaussian distribution in the sampled population. Comparative statistics between ACR and NR groups were analyzed with Student’s *t*-test. P values <0.05 were considered significant. For evaluation of sensitivity and specificity a receiver operating characteristics (ROC) graph was plotted to determine cut off values and to calculate area under the ROC curve (AUC). Pearson’s correlation was used to measure strength of a linear association between two variables and was denoted by r.

## Results

### Altered microRNA levels in the serum of heart-transplanted patients

This study included heart transplant recipients with histologically-verified ACR (n = 26) enrolled as part of the Biomarkers in Transplantation Canada-wide Trial, who underwent heart transplantation between February 2009 and September 2013. This group of patients was compared with a control group of heart transplant recipients without allograft rejection (n = 37) from the same centres and within the same time-period. Detailed patient characteristics are listed in Table 1.

**Table 1:** Clinical characteristics in acute cellular rejection and non-rejection case control groups.

Patient Characteristics	Acute Cellular Rejection (n = 26)	Non Rejection (n = 37)	*p*-value
Recipient age (median, _IQR_ <25–75>)	52 (37–61)	56 (45–61)	0.3
Recipient gender (male, *n* <%>)	17 (65)	29 (76)	0.3
ISHLT Biopsy Grade, n<%> 0R (none) 1R (mild) 2R (moderate)	- 2 (8) 24 (92)	37 (100) - -	
Primary heart disease, *n* <%> Ischemic cardiomyopathy Non-ischemic cardiomyopathy Valvular cardiomyopathy Congenital cardiomyopathy Miscellaneous	7 (27) 14 (54) 2 (8) 1 (4) 2 (8)	16 (42) 21 (55) 0 (0) 1 (3) 0 (0)	0.2 0.9 0.08 0.8 0.08
Time of Biopsy (Days after Tx) (median, _IQR_ <25–75>)	23 (14–105)	37 (20–59)	0.09
Creatinine (mean, umol/L)	105.7	133.2	0.055

**Fig 1:**
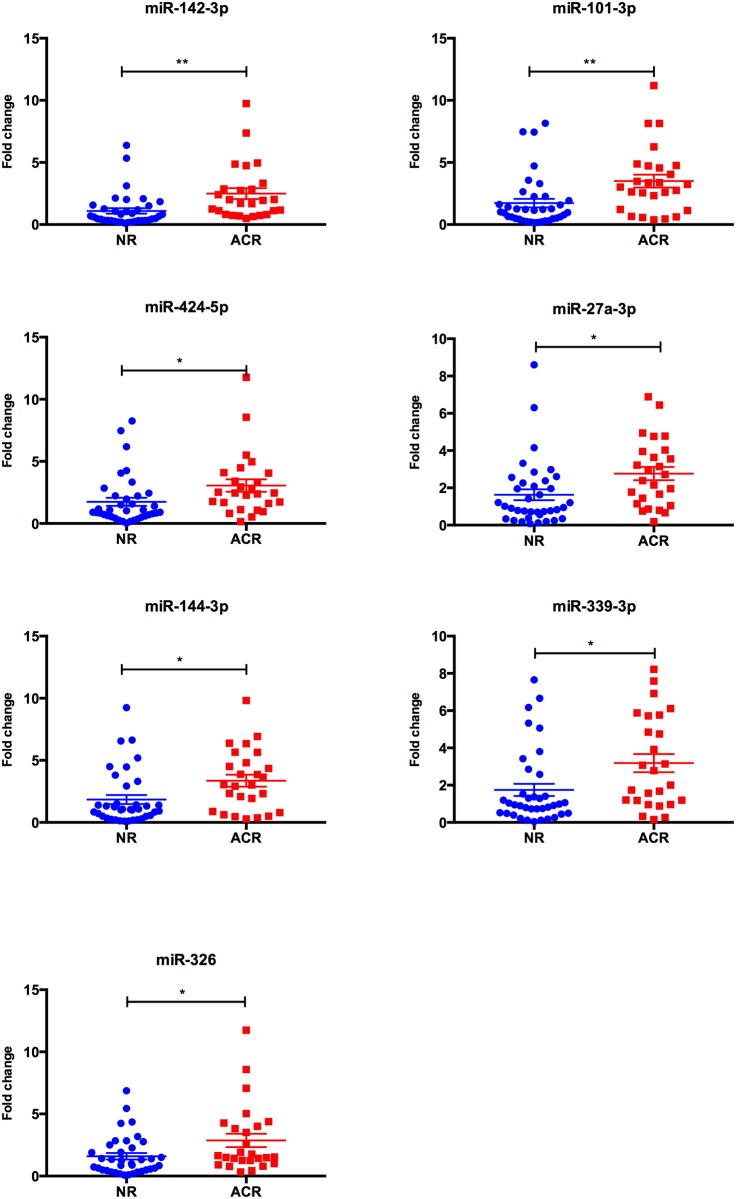
Serum levels of microRNAs in heart transplant patients. The levels of seven microRNAs were significantly higher in heart transplant patients with allograft rejection (ACR, n = 26) compared to the patients without rejection (NR, n = 37). Data is shown as mean ± SEM. Statistical analysis was performed with Student’s *t*-test. *P<0.05; **P<0.01.

We assessed the levels of seven microRNAs (miR-326, miR-142-3p, miR-101, miR-144, miR-27a, miR-424, miR-339-3p), the profiles of which were demonstrated to be altered in our previous study[11], in serum samples from heart transplant patients with and without ACR using a qPCR panel. Using Student’s *t*-test with p = 0.05 as a cutoff value, the levels of all seven microRNAs were significantly higher in the ACR group as compared to the NR group (Fig 1).

### MicroRNA levels in serum discriminate between acute cellular rejection and quiescence

**Fig 2:**
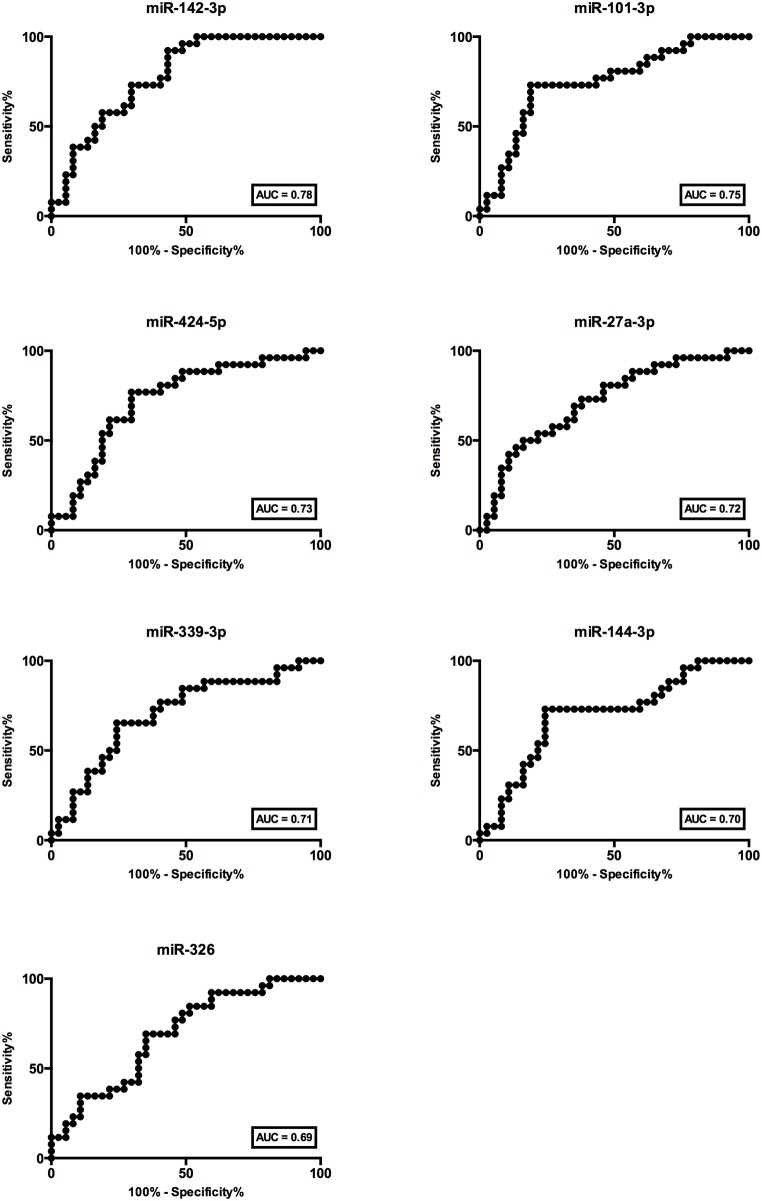
Receiver Operator Characteristic (ROC) analysis. Patients with and without acute cellular rejection could be discriminated by miR-142-3p (AUC = 0.78, CI_95%_ = 0.67 to 0.89), miR-101-3p (AUC = 0.75, CI_95%_ = 0.62 to 0.87), miR-424-5p (AUC = 0.73, CI_95%_ = 0.60 to 0.86), miR-27a-3p (AUC = 0.72, CI_95%_ = 0.59 to 0.85), miR-339-3p (AUC = 0.71, CI_95%_ = 0.57 to 0.84), miR-144-3p (AUC = 0.70, CI_95%_ = 0.56 to 0.83) and miR-326 (AUC = 0.69, CI_95%_ = 0.56 to 0.82).

Receiver operator characteristic (ROC) analysis was performed to evaluate the relationship between sensitivity and specificity based on the relative microRNA levels in cases and controls. All seven microRNA tested could significantly discriminate between ACR and NR, with AUC of 0.78, 0,75, 0.73, 0.72, 0.71, 0.70 and 0.69 for miR-142-3p, miR-101-3p, miR-424-5p, miR-27a-3p, miR-339-3p, miR-144-3p, and miR-326, respectively (Fig 2). However, from the ROC analysis, miR-142-3p and miR-101 have the best diagnostic test performance among the seven microRNAs tested. Therefore, miR-142-3p and miR-101-3p were further investigated in the subsequent analysis.

### MiR-142-3p and miR-101-3p levels stratified by time post-transplantation

**Fig 3:**
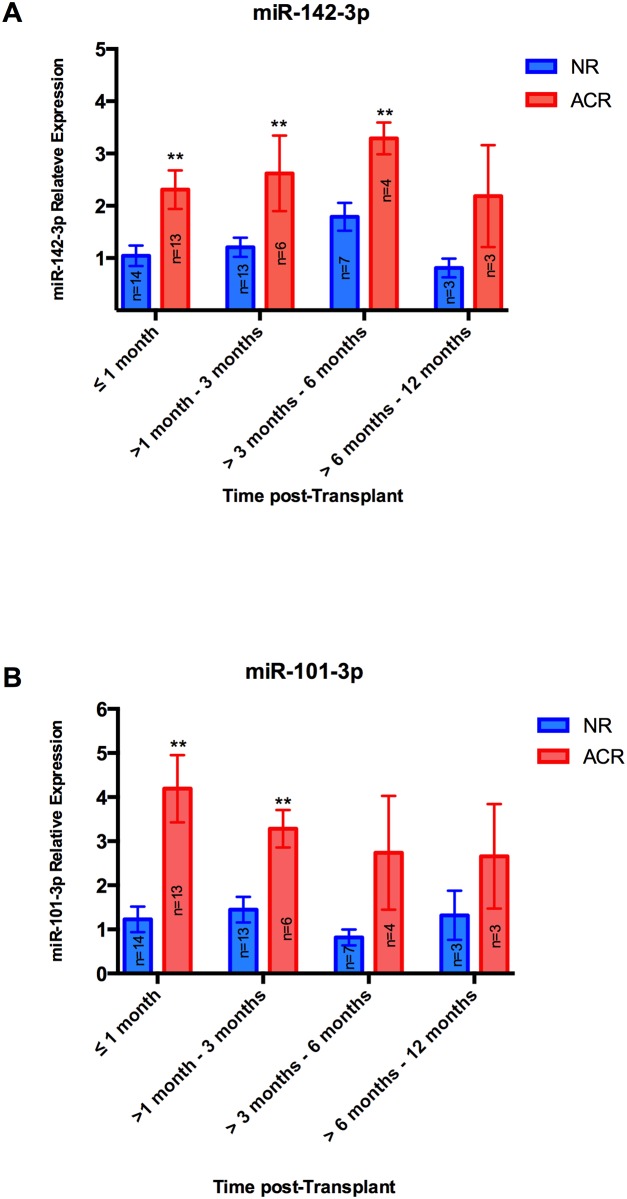
MicroRNA fold change in serum sample of heart transplant patients reported by time post-transplantation. A) MiR-142-3p fold change and B) MiR-101-3p fold change (≤1 month, n = 14(NR) and n = 13(ACR); >1–3 months, n = 13(NR) and n = 6(ACR); >3–6 months, n = 7(NR) and n = 4(ACR); >6–12 months, n = 3(NR) and n = 3(ACR). All data are mean ±SEM, Student’s *t*-test, **p<0.01).

The relative expression of serum miR-142-3p and miR-101-3p levels in serum were analysed over time post-transplantation. Serum microRNA levels were analysed at the time points when endomyocardial biopsy surveillance was performed, i.e. <1 month, 1–3 months, 3–6 months and 6–12 months post transplantation. The serum level of miR-142-3p was significantly higher in ACR patients compared to NR patients for samples collected within 6 months post-transplantation (Fig 3A). For samples collected between 6 months and 1 year post-transplantation, miR-142-3p levels were numerically higher but the difference was not statistically significant. The serum level of miR-101-3p was significantly higher in the ACR group for samples taken in the first 3 months post transplantation, but not in the later time points (Fig 3B). Note that it does not show how the microRNA levels vary over time post transplantation in each patient. Only one result per patient is included for each microRNA. Controls could be attributed to each time period as rejections were diagnosed through the biopsy-screening program.

### Increased serum level of miR-142-3p and miR-101-3p is not indicative of general inflammation

**Fig 4:**
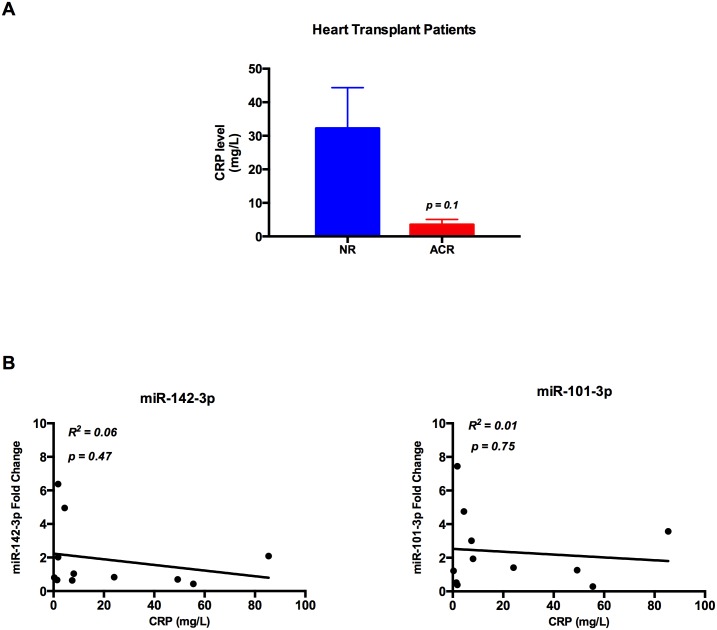
CRP level and microRNAs fold change in heart transplant patients. (A) There is no significant difference between mean CRP level in acute cellular rejection group (n = 4) compared to the non-rejection group (n = 7) of heart transplant patients (NR = 32.2 mg/L vs. ACR = 3.5 mg/L, p = 0.1) and (B) There is no correlation between CRP level in heart transplant patients and miR-142-3p fold change (n = 11; R^2^ = 0.06 and P = 0.47) or miR-101-3p fold change (n = 11; R^2^ = 0.01 and P = 0.75).

To investigate whether the altered serum levels of miR-142-3p and miR-101-3p in heart transplant patients could be a reflection of general inflammation, the correlation between microRNA levels and CRP was performed. First, there was no significant difference in CRP levels between the ACR and NR groups (Fig 4A). Second, there was no correlation between CRP level and miR-142-3p or miR-101-3p levels (Fig 4B).

### Calcineurin inhibitor level does not correlate with miR-142-3p and miR-101-3p in serum

**Fig 5:**
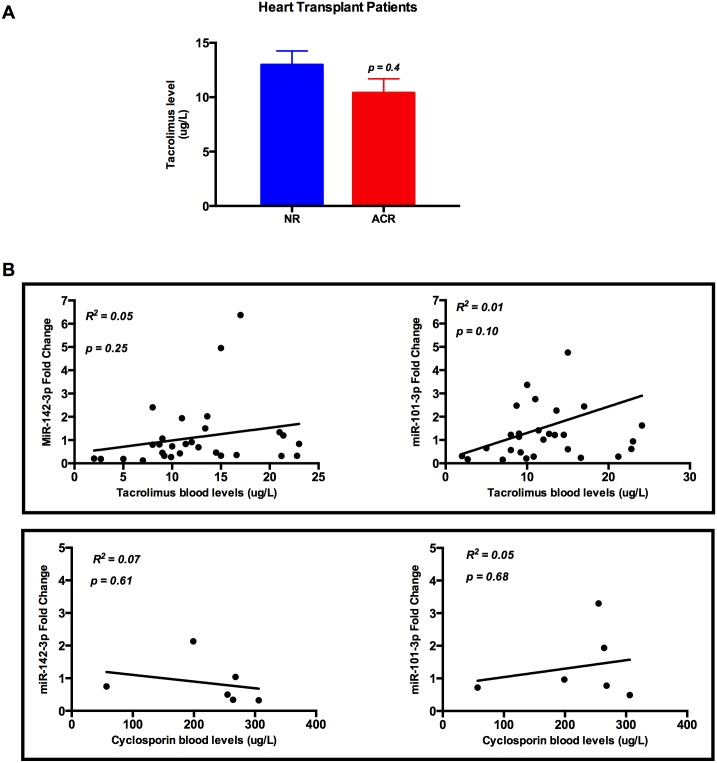
Calcineurin inhibitor levels and microRNA fold change in heart transplant patients. (A) There is no significant difference between mean tacrolimus level in acute cellular rejection group (n = 5) compared to the non-rejection group (n = 24) of heart transplant patients (NR = 12.9 ug/L vs. ACR = 10.4 ug/L, p = 0.4) and (B) There are no correlations between miR-142-3p fold change and tacrolimus (n = 29; R^2^ = 0.05; p = 0.25) or cyclosporin (n = 6; R^2^ = 0.07; p = 0.61) and there are no correlations between miR-101-3p fold change and tacrolimus (n = 29; R^2^ = 0.01; p = 0.10) or cyclosporine (n = 6; R^2^ = 0.05; p = 0.68).

To investigate whether the overall immunosuppression intensity in the heart-transplanted patients could modulate the serum levels of miR-142-3p and miR-101-3p, correlation between the levels of calcineurin inhibitors and miR-142-3p or miR-101-3p in the circulation of heart transplant patients was analysed. There was no significant difference in tacrolimus blood level in ACR group compared to NR group (Fig 5A) and there were no correlations between tacrolimus or cyclosporine levels with either miR-142-3p or miR-101-3p levels in serum (Fig 5B).

### Creatinine levels do not correlate with miR-142-3p and miR-101-3p

**Fig 6:**
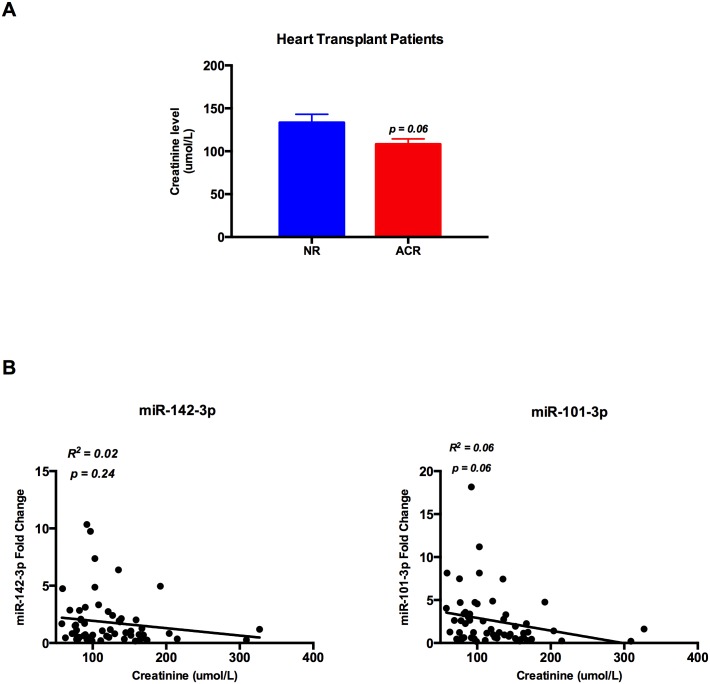
Creatinine level and microRNAs fold change in heart transplant patients. (A) There is no significant difference between mean creatinine level in ACR group (n = 26) vs. NR group (n = 37) in heart transplant patients (NR = 133.2 umol/L vs. ACR = 105.7 umol/L, p = 0.055). (B) There is no correlation between creatinine levels and miR-142-3p (R^2^ = 0.02, p = 0.24) or miR-101-3p (R^2^ = 0.06, p = 0.06) in heart transplant patients.

To assess the relationship between miR-142-3p and miR-101-3p serum levels with kidney function in heart transplant patients, correlation between creatinine levels and miR-142-3p and miR-101-3p was analysed. There was no significant difference in creatinine levels in the ACR group compared to the NR group (Fig 6A) and there was no correlation between either miR-142-3p or miR-101-3p and creatinine levels (Fig 6B).

## Discussion

In heart transplantation, there is an unmet need for non-invasive and relevant diagnostic tools to minimize or even eliminate the use of endomyocardial biopsies. In recent years microRNAs have emerged as promising biomarker candidates in the field of solid organ transplantation given that their expression patterns reflecting both physiological and underlying pathological conditions in humans and their involvement in the regulation of both innate and adaptive immunity[13–16]. The highly tissue-specific expression and remarkable stability in serum and other body fluids[17] are other features, which make microRNAs suitable as biomarkers. Despite the high ribonuclease activity in blood, microRNAs found in circulation are protected from degradation by associating with proteins such as Ago2 or encapsulation in extracellular vesicles[18].

This work is a follow-up study and an independent validation of our previous pilot study[11], using a larger independent cohort with samples collected within the Biomarkers in Transplantation Canada-wide Trial. In our previous study, we profiled the levels of 175 selected serum microRNAs before, during, and after cardiac allograft rejection in a small group of heart transplant patients. The present study validates that seven microRNAs, miR-142-3p, miR-339-3p, miR-326, miR-144-3p, miR-101-3p, miR-27a-3p and miR-424-5p are present in serum samples from heart transplant patients and adequately distinguish ACR from NR. Moreover, in the diagnostic test that combines sensitivity and specificity, the levels of miR-142-3p and miR-101-3p are shown to have the best performance among the seven microRNAs tested that yield AUC-ROC score of 0.78 and 0.75, respectively.

The optimal biomarker of ACR should be able to abolish the need of invasive techniques to monitor and diagnose rejection after organ transplantation. The key elements of evaluating a new biomarker include easy and rapid analysis methods, adding new information upon existing tests and having the potential to change patient management[19,20]. A good biomarker for rejection will not only diminish costs and save patients unnecessary discomfort, but will also be able to identify risk patients already at the time of transplantation and be able to predict an upcoming rejection prior to organ damage. In addition, it could help us the to more accurately titrate the dose of immunosuppressant. However, finding a single biomarker that meets all of these criteria is not likely.

The fact that microRNAs are stable at room temperature, minimally affected by freeze-thawing cycle and may be frozen up to 40 years without any significant degradation [17,21,22], makes them suitable for use in clinical practice where variations in the handling of samples can be expected. Since the levels can be determined with qPCR, a technique used in most clinical laboratories already, the cost of the analysis is predicted to be moderate.

Alternative strategies have been investigated and it is probable that several types of biomarkers will complement each other. AlloMap, a blood-based gene-expression profiling strategy, has been in clinical use for some time. It has been shown to be useful for monitoring rejection and reduce the number of routine biopsies, but can not entirely replace the use of endomyocardial biopsy for screening allograft rejection[5]. In the CARGO and IMAGE studies, an AUC of 0.72 could be shown for AlloMap, with a strong NPV of 98–100%[5,23,24]. The IMAGE study showed non-inferiority of AlloMap compared to endomyocardial biopsy when used between 6 and 60 months post-transplantation. A significant downside to AlloMap is that its reliability is lower <6 months after transplantation[5,25] and it is not useful in the first 2 months post-transplant when most of the ACR episodes occur. In a smaller more recent study, patients did not suffer from an increased number of adverse outcomes when monitored with AlloMap compared to endomyocardial biopsy[26]. Another method for predicting ACR has been suggested where a panel of genomic biomarkers is used to analyse whole blood from the recipient combined with myocardial tissue from the donor heart[27]. This method reached an AUC score of 0.90 for predicting future rejection events when used at the time of transplantation. Hence, in this study, focus lies on predicting future rejections at the time of transplantation and not on diagnosis.

Since AlloMap has had limitations in its ability to distinguish ACR from NR during the first 6 months post-transplantation[5] and is likely dependent on time post-transplantation[24], we wanted to illustrate how microRNA levels react at ACR regardless of the time post-transplantation. In our study, the patients underwent endomyocardial biopsy surveillance according to the following schedule: every week in the first month, once in 2 weeks between 1–3 month, once a month between 3–6 month and once in 2 month between 6–12 month. The serum level of miR-142-3p was significantly higher in ACR patients compared to NR patients in the first 6 months post-transplantation, the period when the overall risk of rejection is highest, which suggests that serum level of miR-142-3p may be useful to diagnose ACR during the first 6 months post transplantation. The performance of miR-101-3p, however, suggested that miR-101-3p level was significantly higher in ACR group compared to NR group only in the first 3 months post-transplantation.

There is always a risk that biomarkers are altered by other factors than the one of interest. We have adressed the question of variability with kidney function, CRP levels and concentrations of immunosuppressants. These results are encouraging as there is no association between either of these factors and the levels of miR-142-3p and miR-101-3p. There are 3 patients in this study that have developed post heart transplantation Cytomegalovirus (CMV) infection at the time of ACR. The fact that there is no correlation between microRNA levels and CRP in this study indicates that microRNA levels are not altered by infection. However, this question needs to be evaluated in further studies due to the limitation of low sample numbers in each group.

MiR-142-3p is a hematopoietic-tissue specific microRNA, whose level can be measured in human serum using qPCR assay. MiR-142-3p is highly expressed in T lymphocytes, the main player in ACR, and has been shown to play a role in regulatory T cells and in promoting tolerance in solid organ transplantation[28,29]. Related to allograft rejection, the alteration of miR-142-3p expression profile in the biopsy samples of ACR has been demonstrated both in human and animal model[29–32]. The fact that miR-142-3p originates from immune cells, not from the graft tissue, raise the possibility to predict rejection prior to organ damage. Mir-101-3p, on the other hand, has not been well characterized in connection with transplant immunity, but it has been linked to an altered microRNA profile during allograft rejection of liver transplantation in an animal model[33], suggesting the possibility that allograft rejection of different solid organ transplantation may share the same signalling pathways.

Several limitations need to be considered in interpreting the results of this study, mainly due to the modest size and the limitation of the validation cohort in this study that needs to be further validated in a multicenter setting.

In summary, our study shows that miR-142-3p and miR-101-3p can accurately discriminate heart transplant patients with ACR from those with no rejection. We suggest that they are promising serum biomarkers for non-invasive surveillance of rejection post transplantation.

## Supporting Information

S1 Fig Mean Ct value of 7 microRNAs tested. (PDF)

S1 Table ROC analysis of miR-142-3p. (PDF)

S2 Table ROC analysis of miR-101-3p. (PDF)

S3 Table CRP level (mg/L) in NR vs. ACR groups. (PDF)

S4 Table Tacrolimus level (ug/L) in NR vs. ACR groups. (PDF)

S5 Table Creatinine level (umol/L) in NR vs. ACR groups. (PDF)
