# Supplementary figures and images for: Association of Serum MiR-142-3p and MiR-101-3p Levels with Acute Cellular Rejection after Heart Transplantation
Source: PLoS One. 2017 Jan 26;12(1):e0170842. doi: 10.1371/journal.pone.0170842 (PMC5268768; doi:10.1371/journal.pone.0170842)

**S1 Fig. Mean Ct value of 7 microRNAs tested**

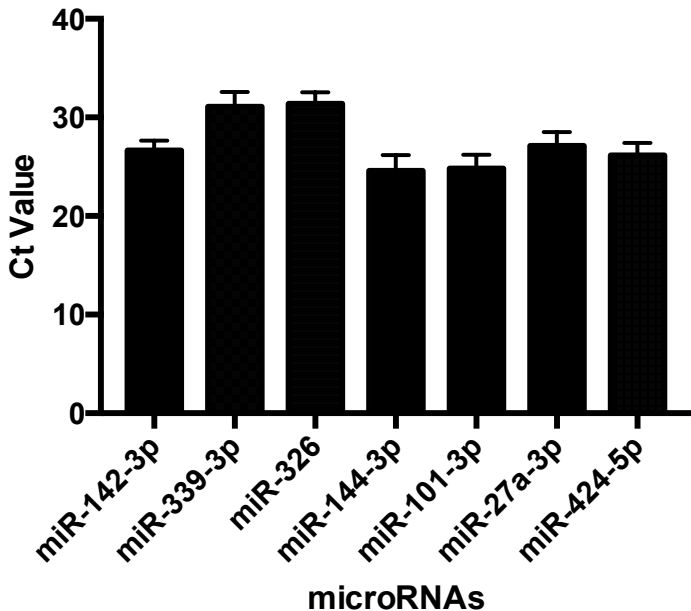

Supplement: S1 Fig — (PDF) [file pone.0170842.s001.pdf]
