# Supplementary material for: Association of Serum MiR-142-3p and MiR-101-3p Levels with Acute Cellular Rejection after Heart Transplantation
Source: PLoS One. 2017 Jan 26;12(1):e0170842. doi: 10.1371/journal.pone.0170842 (PMC5268768; doi:10.1371/journal.pone.0170842)
Supplement: S1 Table — (PDF) [file pone.0170842.s002.pdf]

S1 Table. ROC analysis of miR-142-3p

Fold Change	Sensitivity%	95% CI	Specificity%	95% CI
> 0.1454	100	86.77% to 100%	2.703	0.0684% to 14.16%
> 0.178	100	86.77% to 100%	5.405	0.6615% to 18.19%
> 0.1896	100	86.77% to 100%	8.108	1.704% to 21.91%
> 0.1997	100	86.77% to 100%	10.81	3.025% to 25.42%
> 0.2316	100	86.77% to 100%	13.51	4.537% to 28.77%
> 0.2638	100	86.77% to 100%	16.22	6.193% to 32.01%
> 0.2957	100	86.77% to 100%	18.92	7.962% to 35.16%
> 0.3207	100	86.77% to 100%	21.62	9.827% to 38.21%
> 0.3239	100	86.77% to 100%	24.32	11.77% to 41.2%
> 0.3253	100	86.77% to 100%	27.03	13.79% to 44.12%
> 0.3269	100	86.77% to 100%	29.73	15.87% to 46.98%
> 0.3325	100	86.77% to 100%	32.43	18.01% to 49.79%
> 0.3486	100	86.77% to 100%	35.14	20.21% to 52.54%
> 0.3928	100	86.77% to 100%	37.84	22.46% to 55.24%
> 0.4391	100	86.77% to 100%	40.54	24.75% to 57.9%
> 0.4576	100	86.77% to 100%	43.24	27.1% to 60.51%
> 0.4761	100	86.77% to 100%	45.95	29.49% to 63.08%
> 0.4941	96.15	80.36% to 99.9%	45.95	29.49% to 63.08%
> 0.5502	96.15	80.36% to 99.9%	48.65	31.92% to 65.6%
> 0.6203	96.15	80.36% to 99.9%	51.35	34.4% to 68.08%
> 0.652	92.31	74.87% to 99.05%	51.35	34.4% to 68.08%
> 0.6783	92.31	74.87% to 99.05%	54.05	36.92% to 70.51%
> 0.6929	92.31	74.87% to 99.05%	56.76	39.49% to 72.9%
> 0.7102	88.46	69.85% to 97.55%	56.76	39.49% to 72.9%
> 0.7291	84.62	65.13% to 95.64%	56.76	39.49% to 72.9%
> 0.768	80.77	60.65% to 93.45%	56.76	39.49% to 72.9%
> 0.8102	76.92	56.35% to 91.03%	56.76	39.49% to 72.9%
> 0.819	76.92	56.35% to 91.03%	59.46	42.1% to 75.25%
> 0.8257	73.08	52.21% to 88.43%	59.46	42.1% to 75.25%
> 0.8697	73.08	52.21% to 88.43%	62.16	44.76% to 77.54%
> 0.9747	73.08	52.21% to 88.43%	64.86	47.46% to 79.79%
> 1.051	73.08	52.21% to 88.43%	67.57	50.21% to 81.99%
> 1.091	73.08	52.21% to 88.43%	70.27	53.02% to 84.13%
> 1.12	69.23	48.21% to 85.67%	70.27	53.02% to 84.13%
> 1.145	65.38	44.33% to 82.79%	70.27	53.02% to 84.13%
> 1.182	61.54	40.57% to 79.77%	70.27	53.02% to 84.13%
> 1.227	61.54	40.57% to 79.77%	72.97	55.88% to 86.21%
> 1.261	57.69	36.92% to 76.65%	72.97	55.88% to 86.21%
> 1.386	57.69	36.92% to 76.65%	75.68	58.8% to 88.23%
> 1.54	57.69	36.92% to 76.65%	78.38	61.79% to 90.17%
> 1.627	57.69	36.92% to 76.65%	81.08	64.84% to 92.04%
> 1.693	53.85	33.37% to 73.41%	81.08	64.84% to 92.04%
> 1.772	50	29.93% to 70.07%	81.08	64.84% to 92.04%
> 1.89	50	29.93% to 70.07%	83.78	67.99% to 93.81%
> 1.973	46.15	26.59% to 66.63%	83.78	67.99% to 93.81%
> 2.012	42.31	23.35% to 63.08%	83.78	67.99% to 93.81%
> 2.021	42.31	23.35% to 63.08%	86.49	71.23% to 95.46%
> 2.053	38.46	20.23% to 59.43%	86.49	71.23% to 95.46%
> 2.108	38.46	20.23% to 59.43%	89.19	74.58% to 96.97%
> 2.27	38.46	20.23% to 59.43%	91.89	78.09% to 98.3%
> 2.577	34.62	17.21% to 55.67%	91.89	78.09% to 98.3%
> 2.797	30.77	14.33% to 51.79%	91.89	78.09% to 98.3%
> 2.857	26.92	11.57% to 47.79%	91.89	78.09% to 98.3%
> 2.991	23.08	8.974% to 43.65%	91.89	78.09% to 98.3%
> 3.223	23.08	8.974% to 43.65%	94.59	81.81% to 99.34%
> 4.038	19.23	6.555% to 39.35%	94.59	81.81% to 99.34%
> 4.809	15.38	4.356% to 34.87%	94.59	81.81% to 99.34%
> 4.913	11.54	2.446% to 30.15%	94.59	81.81% to 99.34%
> 5.147	7.692	0.9455% to 25.13%	94.59	81.81% to 99.34%
> 5.857	7.692	0.9455% to 25.13%	97.3	85.84% to 99.93%
> 6.873	7.692	0.9455% to 25.13%	100	90.51% to 100%
> 8.561	3.846	0.09733% to 19.64%	100	90.51% to 100%
