# Supplementary material for: Association of Serum MiR-142-3p and MiR-101-3p Levels with Acute Cellular Rejection after Heart Transplantation
Source: PLoS One. 2017 Jan 26;12(1):e0170842. doi: 10.1371/journal.pone.0170842 (PMC5268768; doi:10.1371/journal.pone.0170842)
Supplement: S3 Table — (PDF) [file pone.0170842.s004.pdf]

S3 Table. CRP level (mg/L) in NR vs. ACR groups

NR	ACR
55.5	0.3
1.4	7.4
85.4	4.4
24.1	1.8
1.8	
49.3	
8	
