# Supplementary material for: Association of Serum MiR-142-3p and MiR-101-3p Levels with Acute Cellular Rejection after Heart Transplantation
Source: PLoS One. 2017 Jan 26;12(1):e0170842. doi: 10.1371/journal.pone.0170842 (PMC5268768; doi:10.1371/journal.pone.0170842)
Supplement: S4 Table — (PDF) [file pone.0170842.s005.pdf]

S4 Table. Tacrolimus level (ug/L) in NR vs. ACR groups

NR	ACR
11.4	8
13.6	10
10.8	15
17	8
9.9	11
12.7	
13.4	
14.5	
21	
21.2	
8.7	
16.6	
2	
2.7	
24.1	
22.8	
9.2	
5	
7	
12	
23	
15	
9	
9	
