# Supplementary material for: Association of Serum MiR-142-3p and MiR-101-3p Levels with Acute Cellular Rejection after Heart Transplantation
Source: PLoS One. 2017 Jan 26;12(1):e0170842. doi: 10.1371/journal.pone.0170842 (PMC5268768; doi:10.1371/journal.pone.0170842)
Supplement: S5 Table — (PDF) [file pone.0170842.s006.pdf]

S5 Table. Creatinine level (umol/L) in NR vs. ACR groups

NR	ACR
147	130
174	90
152	97
84	88
167	163
204	121
84	108
168	103
135	192
309	159
90	127
169	136
76	59
152	89
143	100
122	82
95	124
96	72
92	103
139	58
78	78
76	82
215	96
111	137
99	116
327	103
77	
80	
78	
158	
119	
163	
145	
137	
92	
63	
113	
